# *H3F3A* mutant allele specific imbalance in an aggressive subtype of diffuse midline glioma, H3 K27M-mutant

**DOI:** 10.1186/s40478-020-0882-4

**Published:** 2020-02-05

**Authors:** Sachi Maeda, Fumiharu Ohka, Yusuke Okuno, Kosuke Aoki, Kazuya Motomura, Kazuhito Takeuchi, Hironao Kusakari, Nobuyuki Yanagisawa, Shinya Sato, Junya Yamaguchi, Kuniaki Tanahashi, Masaki Hirano, Akira Kato, Hiroyuki Shimizu, Yotaro Kitano, Shintaro Yamazaki, Shinji Yamashita, Hideo Takeshima, Keiko Shinjo, Yutaka Kondo, Toshihiko Wakabayashi, Atsushi Natsume

**Affiliations:** 1 Department of Neurosurgery, Nagoya University Graduate School of Medicine, 65 Tsurumai-cho, Showa-ku, Nagoya, 466-8550 Japan; 2 Medical Genomics Center, Nagoya University Hospital, 65 Tsurumai-cho, Showa-ku, Nagoya, 466-8550 Japan; 3 Department of Pathology, St. Marianna University School of Medicine Yokohama City Seibu Hospital, 1197-1 Yasashi-cho, Asahi-ku, Yokohama, 241-0811 Japan; 4 Molecular Pathology and Genetics Division, Kanagawa Cancer Center Research Institute, 2-3-2 Nakao, Asahi-ku, Yokohama, 241-8515 Japan; 5 Department of Neurosurgery, Division of Clinical Neuroscience, Faculty of Medicine, University of Miyazaki, 5200, Kiyotake-cho, Kihara, Miyazaki, 889-1601 Japan; 6 Division of Cancer Biology, Nagoya University Graduate School of Medicine, Nagoya, Japan, 65 Tsurumai-cho, Showa-ku, Nagoya, 466-8550 Japan

**Keywords:** Diffuse midline glioma, H3 K27M-mutant, Mutant allele specific imbalance, Whole-genome sequencing, Droplet digital polymerase chain reaction

## Abstract

Diffuse midline glioma, H3 K27M-mutant is a lethal brain tumor located in the thalamus, brain stem, or spinal cord. H3 K27M encoded by the mutation of a histone H3 gene such as *H3F3A* plays a pivotal role in the tumorigenesis of this type of glioma. Although several studies have revealed comprehensive genetic and epigenetic profiling, the prognostic factors of these tumors have not been identified to date. In various cancers, oncogenic driver genes have been found to exhibit characteristic copy number alterations termed mutant allele specific imbalance (MASI). Here, we showed that several diffuse midline glioma, H3 K27M-mutant exhibited high variant allele frequency (VAF) of the mutated *H3F3A* gene using droplet digital polymerase chain reaction (ddPCR) assays. Whole-genome sequencing (WGS) revealed that these cases had various copy number alterations that affected the mutant and/or wild-type alleles of the *H3F3A* gene. We also found that these MASI cases showed a significantly higher Ki-67 index and poorer survival compared with those in the lower VAF cases (*P* < 0.05). Our results indicated that the MASI of the *H3F3A K27M* mutation was associated with the aggressive phenotype of the diffuse midline glioma, H3 K27M-mutant via upregulation of the H3 K27M mutant protein, resulting in downregulation of H3K27me3 modification.

## Introduction

Diffuse midline glioma is an infiltrative glial neoplasm located in the thalamus, brain stem, or spinal cord. Most of these tumors develop in children, adolescents, and young adults with a lethal clinical course. Most cases cannot achieve maximum tumor removal owing to tumor location and effective adjuvant therapies other than radiation therapy have not been identified to date. Recent comprehensive molecular analyses revealed that the Lys 27-to-methionine (K27M) mutations at one allele of the histone H3 gene such as the *H3F3A* gene were found in approximately 80% of diffuse intrinsic pontine gliomas (DIPGs), 50% of thalamic tumors, and 60% of spinal tumors [1–6]. Diffuse midline glioma exhibiting heterozygous H3 K27M mutation is defined as diffuse midline glioma, H3 K27M-mutant by the 2016 World Health Organization Classification of Tumors of the Central Nervous System [7]. H3 K27M protein encoded by the H3 K27M mutated allele of this gene plays a pivotal role in tumor formation via the global loss of the H3K27me3 level [3, 8–10]. Molecular mechanisms during diffuse midline glioma, H3 K27M-mutant formation have been well-studied; however, the prognostic markers of this type of glioma have not been identified to date.

Several studies have reported that various cancers exhibit mutant allele specific imbalance (MASI) of driver oncogenes [11–16]. The copy number gain of the mutant allele and/or loss of the wild-type allele of these genes constitutes MASI. In *KRAS*-mutant pancreatic adenocarcinomas and colorectal cancers, MASI of the *KRAS* gene has been associated with a poorer prognosis compared with tumors with heterozygous *KRAS* mutations [17]. The MASI of the driver oncogene might be a new class of potential biomarkers for malignancy, even though the MASI of the *H3F3A* gene in the diffuse midline glioma, H3 K27M-mutant has not been identified yet.

In the present study, we found that a subset of the diffuse midline glioma, H3 K27M-mutant exhibited high variant allele frequency (VAF) of *H3F3A K27M* mutation using droplet digital polymerase chain reaction (ddPCR). Whole-genome sequencing (WGS) revealed the MASI of the *H3F3A K27M* mutation in these cases. We found that MASI cases exhibited a significantly higher Ki-67 index and poorer survival together with a lower H3K27me3 level compared with lower VAF cases. These data suggest that the MASI of *H3F3A K27M* mutation contributed to an aggressive phenotype of the diffuse midline glioma, H3 K27M-mutant.

## Materials and methods

### DNA extraction from tumor and blood samples

Tumor samples were obtained intra-operatively. DNA was extracted from frozen tumor and blood samples using a QIAamp DNA Mini Kit (Qiagen, Hilden, Germany) according to the manufacturer’s instructions. The amount of obtained DNA was evaluated by the Qubit dsDNA HS Assay Kit (Invitrogen, Paisley, Scotland). We also extracted DNA from formalin-fixed paraffin-embedded (FFPE) tumor samples using the GeneRead DNA FFPE Kit (Qiagen) according to the manufacturer’s instructions.

### Sanger sequencing

We performed Sanger sequencing for the detection of *H3F3A* gene mutations in DNA extracted from the tumor samples. We amplified a 194-base pair (bp) fragment for DNA spanning the sequence encoding histone H3 Lysine (K) 27 of the *H3F3A* gene. We applied a conventional PCR involving the following steps: 35 cycles with denaturation at 98 °C for 10 s, annealing at 55 °C for 30 s, and extension at 68 °C for 30 s, with a final extension step at 68 °C for 5 min; the forward primer (5′-TGCTGGTAGGTAAGTAAGGAG-3′) and reverse primer (5′-AGCAGTAGTTAAGTGTTCAAATG-3′) were used. Sequence analysis was performed by ApE v2.0.55.

### Droplet digital PCR (ddPCR)

To evaluate the VAF of the *H3F3A K27M* mutation, we performed ddPCR on all samples. The reaction mixture for ddPCR comprised 10 μl of 2 × ddPCR Supermix for probes (UniqueAssayID: dHsaMDV2510510; Bio-Rad Laboratories, Pleasanton, CA, USA), and 1 μl of 20 × target (FAM) and wild-type (HEX) probe with 30 ng of DNA from each tumor specimen. Each mixture was mixed with 65 μl of Droplet Generation Oil (Bio-Rad Laboratories) and droplets were generated using the QX200 Droplet Generator (Bio-Rad Laboratories). PCR amplification was performed on a C1000 thermo cycler (Bio-Rad Laboratories) with the following thermal cycling conditions: 40 cycles of denaturation of 94 °C for 30 s, annealing and extension of 55 °C for 60 s with a ramp rate of 2 °C/s. Next, the fluorescence intensity of each droplet was calculated using a QX200 Droplet Reader (Bio-Rad Laboratories) and analyzed using QuantaSoft droplet reader software (Bio-Rad Laboratories). VAF was calculated by the number of positive dots of FAM probes/(those of FAM + those of HEX).

### Construction of H3F3A wild-type and K27M mutant plasmid

To validate VAF values measured by ddPCR, we constructed a pcDNA3.0 plasmid containing each wild-type *H3F3A* and *H3F3A K27M*. The construct of wild-type *H3F3A* and *H3F3A K27M* sequences were obtained by PCR amplification using the DNA of each case. The forward primer (5′-CGAAGCTTAGGCCGTTCGAGGTAATTTTT-3′) and reverse primer (5′-ATGGATCCGGTCTCCTTAGACCTCCAGGTAA-3′) were used. The amplified PCR product was digested with *Bam*HI and *Hind*III and ligated into a multi-cloning site of pcDNA3.0 vector using the Ligation Mix (Takara bio, Shiga, Japan).

### Immunohistochemistry (IHC)

FFPE tissue was sectioned and deparaffinized and antigen retrieval was performed by microwave oven heating in sodium citrate buffer (10 mM, pH 6.0) at 95 °C for 20 min. To block endogenous peroxidase, slides were dipped in 3% H_2_O_2_ with methanol for 10 min at room temperature. The slides were subsequently blocked in Dako Antibody Diluent (Dako, Glostrup, Denmark) for 10 min at room temperature. The primary antibodies were diluted in blocking buffer and incubated overnight at 4 °C. We used rabbit monoclonal anti-histone H3 K27M antibody (RevMAb Biosciences, San Francisco, CA, USA), mouse monoclonal anti-histone H4 antibody (Cell signaling, Beverly, MA), rabbit monoclonal anti-trimethyl-histone H3 (Lys27) antibody (Merck Millipore, Darmstadt, Germany), and mouse anti-Ki-67 antigen antibody (Dako). Secondary antibodies were goat anti-rabbit IgG (H + L) highly cross-absorbed secondary antibody, Alexa fluor 546 (Thermo Fisher Scientific, Waltham, MA, USA), or goat anti-mouse IgG (H + L) highly cross-absorbed secondary antibody, Alexa fluor 488 (Thermo Fisher Scientific). Slides were stained with DAPI using VECTASHIELD mounting medium with DAPI (Vector Laboratories, Burlingame, CA). A BZ-X710 microscope (Keyence, Osaka, Japan) was used for fluorescence microscopy.

### Image analysis of IHC staining of tumor sections

For image analyses upon IHC staining of the samples, all the images were obtained using appropriate conditions and thus, consistent findings were obtained for various control samples. The tumor content of the tumor specimens was calculated as H3 K27M positive cell numbers/DAPI positive cell numbers. The frequency of Ki-67 positive cells in tumor cells was calculated as Ki-67 positive cell numbers/H3 K27M positive cell numbers. The frequency of H3K27me3 positive cells was calculated as the H3K27me3 positive cell numbers/histone H4 positive cell numbers. The fluorescent intensity of H3 K27M and histone H4 was quantified by converting intensity to a gray scale based on the RGB color model using Adobe Photoshop CS6 (Adobe Systems Incorporated, San Jose, CA). The relative fluorescence intensity of H3 K27M was calculated as the florescence intensity of H3 K27M/florescence intensity of histone H4 in each tumor cell. Median or mean values of these evaluated values were calculated among those obtained in four fields.

### Whole-genome sequencing (WGS)

DNA libraries were prepared using TruSeq DNA PCR-Free Library Prep Kit (Illumina, San Diego, CA, USA) according to the manufacturer’s instructions. The NovaSeq6000 platform (Illumina) was used to sequence and generate 400-bp long paired-end reads. A median of 717,605,970 reads per sample was obtained and aligned to cover the hg19 reference genome with 33.2x coverage using the Burrows-Wheeler aligner (http://bio-bwa.sourceforge.net/) with default parameters and a -mem option. Sequence variations were detected and annotated using VarScan2 (http://dkoboldt.github.io/varscan/). For copy number analysis, the coverage of each 10 kb span was compared with the mean coverage of the entire genome. The data was segmented by circular binary segmentation using DNAcopy 1.56.0 with the modified parameters [18]. We also evaluated the mismatch ratio of the common single nucleotide polymorphisms (SNPs), which were reported to be found with more than 5% frequency. The moving average of three sequential median values of mismatch ratio of SNPs located on each 10 kb region was calculated. B allele frequency (BAF) was calculated by the moving average of mismatch ratio and (1-moving average of mismatch ratio) in each SNP region. Higher or lower values between the moving average of the mismatch ratio and (1-moving average of mismatch ratio) were described as yellow or green dots, respectively.

### Targeted-capture sequencing

Sequencing libraries were prepared according to the manufacturer’s protocol using a SureSelect XT target enrichment system and ClearSeq SS Comprehensive Cancer bait that targeted 151 cancer-associated genes (Agilent technologies, Santa Clara, CA). Prepared libraries were run on a Miseq next-generation sequencing platform using Miseq Reagent kit v3 (Illumina). The sequenced reads were aligned to the hg19 reference genome using the Burrows Wheeler aligner with default parameters and a -mem option. PCR duplicates were removed using Picard tools (http://broadinstitute.github.io/picard/). Sequence variations were detected and annotated using VarScan2 and the variants that fulfilled the following criteria were adopted for further analyses: (i) number of variant reads > 10, and (ii) variants allele frequency ≥ 0.05. The adopted variants were annotated with ANNOVAR (http://annovar.openbioinformatics.org/en/latest/) and the variants that met at least one of the following criteria were excluded from the analyses: (i) synonymous and ambiguously annotated (unknown) variants, (ii) common SNPs which were defined as those with ≥1% allele frequency in the Exome Aggregation Consortium (ExAC) (http://exac.broadinstitute.org/), the 1000 Genomes Project (http://www.internationalgenome.org/), and National Heart, Lung, and Blood Institute (NHLBI) Exome Sequencing Project (ESP) 6500 (https://evs.gs.washington.edu/EVS/) or registered in NCBI dbSNP build 138 (https://www.ncbi.nlm.nih.gov/snp/).

### Fluorescence in situ hybridization (FISH)

FISH assay was performed according to the manufacturer’s instructions with minor modifications. Briefly, the FFPE tissue sections were deparaffinized with xylene. These samples were then pretreated using pretreatment buffer supplied in the Vysis paraffin Pretreatment IV & Post-hybridization Wash Buffer kit (Abbott laboratories, Chicago, IL). Aquarius® Pathology Probes 1p36/1q25 (Riken genesis CO., LTD. Tokyo, Japan) were applied to the pretreated sections. Samples and probes were denatured at 80 °C for 3 min prior to the application, followed by a hybridization step at 37 °C for 48 h. Then, the sections were subjected to the washing step using wash buffer supplied in the Vysis paraffin Pretreatment IV & Post-hybridization Wash Buffer kit (Abbott laboratories). The sections were then stained with DAPI, mounted on a slide with mounting medium (Riken genesis CO., TLD.) and were further assessed under a Nikon fluorescent microscope with appropriate filters (Nikon instruments, Tokyo, Japan).

### Western blot analysis

Cell lysate was extracted from FFPE tissue samples using a Qproteome FFPE Tissue Kit (Qiagen). 15 μg of total protein from each sample was run on 4–12% bis-tris precast polyacrylamide gel (Thermo Fisher Scientific), transferred to PVDF membrane using a wet system and probed overnight with primary antibody. The following primary antibodies were used: rabbit polyclonal anti-Histone H3 antibody (Abcam, Cambridge, MA), rabbit monoclonal anti-histone H3 K27M (RevMAb Biosciences) and rabbit monoclonal anti-trimethyl-histone H3 (Lys27) antibody (Merck Millipore). The blot was then probed with the rabbit HRP-conjugated secondary antibodies (GE Healthcare, Buckinghamshire, UK). Membranes were revealed using chemiluminescent detection reagent (GE Healthcare) and enhanced signal was detected in CCD imaging system. Protein extracted from the FFPE tumor sample of *H3F3A* wild-type glioma (*IDH1* mutated) case was used as a negative control for H3 K27M and as a positive control for H3K27me3 [19].

### Statistical analysis

The statistical significance of differences between two groups was analyzed by paired Student t-test, Wilcoxon signed-rank test, and Wilcoxon rank-sum test with R Statistical Software (version 3.6.1; R Foundation for Statistical Computing, Vienna, Austria). Overall survival (OS) was calculated as the time of tumor removal until death or last follow-up. Progression-free survival (PFS) was calculated as the time of tumor removal until recurrence or last follow-up. OS and PFS were evaluated using a log-rank test and Cox proportional hazards regression modeling. All reported *P* values were two-sided, with *P* < 0.05 considered statistically significant.

## Results

### DdPCR analyses of H3F3A K27M mutation

**Fig. 1:**
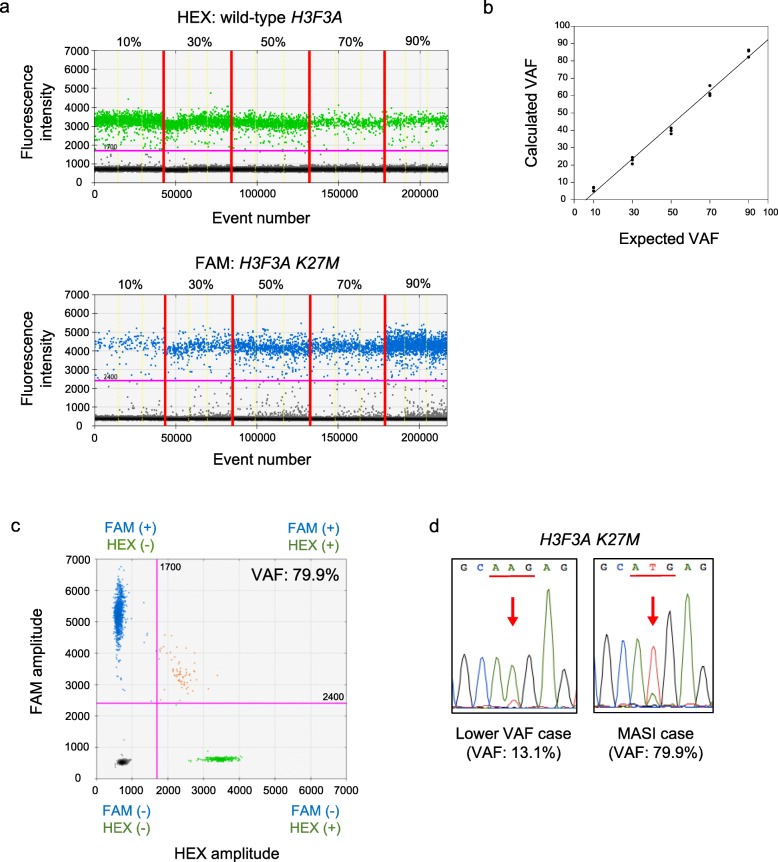
Genetic analyses of *H3F3A* gene mutation using ddPCR and Sanger sequencing. **a** Results of ddPCR using various mixture DNA consisting of *H3F3A K27M* and wild-type *H3F3A*. Upper dot plots indicate wild-type *H3F3A* DNA dots (green) and lower dot plots indicate *H3F3A K27M* DNA dots (blue). The x-axis is the number of droplets including PCR products. The y-axis shows the intensity of HEX and FAM. The pink line shows the threshold (HEX: 1700, FAM: 2400) **b** Correlation of VAFs calculated by ddPCR with expected VAF. The x-axis is the expected VAF and the y-axis is VAF calculated by ddPCR (*P* < 0.01, r^2^ = 0.99). **c** 2D cluster plot of droplet fluorescence of *H3F3A K27M* and wild-type *H3F3A* dots in more than 50% VAF of *H3F3A K27M* case (case 12). FAM positive and HEX negative droplets (blue) include *H3F3A K27M*. HEX positive and FAM negative droplets (green) include wild-type *H3F3A*. VAF of *H3F3A K27M* was calculated as (blue dot numbers/blue dot numbers + green dot numbers). **d** Sanger sequencing of lower than 50% VAF case (left; 13.1% of VAF; case 8) or more than 50% VAF case (right; 79.9% of VAF; case 12). Hot spots of *H3F3A K27M* mutation are indicated by the red arrows

DdPCR calculated the VAF of the *H3F3A K27M* mutated genes. To analyze the correlation between the calculated VAF with ddPCR and the expected VAF, we performed ddPCR with a serial proportional mixture of plasmid DNAs containing the *H3F3A K27M* mutated sequence and those of the *H3F3A* wild-type (Fig. 1a). We detected a significant correlation between the calculated VAF and expected VAF (*P* < 0.01; *r*^*2*^ = 0.99) (Fig. 1b). Using this assay, we analyzed 15 cases of diffuse midline glioma, H3 K27M-mutant (Table 1). Four out of 15 diffuse midline glioma, H3 K27M-mutant cases exhibited more than 50% VAF (Fig. 1c and Table 1). Sanger sequencing also revealed a higher mutant peak than that of the wild-type allele in these four cases (Fig. 1d). DdPCR revealed that three (case 10, 12 and 15) out of the four cases whose blood samples were available exhibited no germline *H3F3A K27M* mutations. These data indicated that the four cases might exhibit MASI of *H3F3A K27M* mutation. Therefore, we performed WGS using DNA derived from the four cases that exhibited more than 50% VAF.

**Table 1:** Summary of clinical features, VAF of *H3F3A K27M* and relative H3 K27M fluorescence intensity

Case Number	Age at onset	Sex	Tumor location	VAF of *H3F3A K27M* (%)	Relative H3 K27M fluorescence intensity(Median)
1	6	F	Pons	40.7	^a^N/A
2	30	F	Insular gyrus	42.4	1.13
3	53	M	Brainstem, Temporal lobe	0.8	0.23
4	14	M	Spinal Cord	38.3	1.27
5	37	M	Brainstem	73.2	1.80
6	37	F	Thalamus, Ventricle, Midbrain	40.7	1.07
7	15	F	Basal ganglia	29.6	^a^N/A
8	29	M	Thalamus	13.1	0.52
9	31	F	Thalamus	42.8	^a^N/A
10	14	M	Spinal Cord	68.0	1.98
11	9	F	Brainstem	22.7	0.81
12	31	F	Brainstem, Thalamus, Cerebellum	79.9	1.50
13	14	M	Thalamus	49.6	^a^N/A
14	24	M	Corpus callosum	43.0	^a^N/A
15	21	M	Corpus callosum	65.9	1.09

^a^*N/A* not available

### MASI of H3F3A K27M mutation identified by WGS

**Fig. 2:**
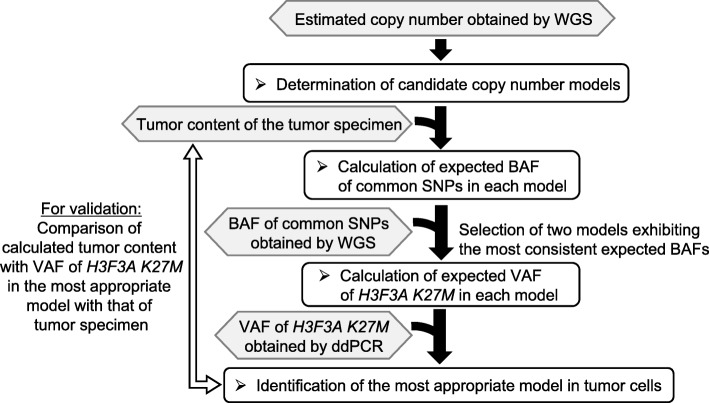
Flowchart for the identification of the most appropriate chromosomal structure model. Using the total copy number of chromosome 1 obtained by WGS, tumor content of the tumor specimen (H3 K27M positive cells/DAPI positive cells in IHC), BAF of common SNPs obtained by WGS, and VAF of *H3F3A K27M* obtained by ddPCR, we identified the most consistent chromosomal structure model. Then, we calculated the tumor content with VAF of *H3F3A K27M* in the most appropriate model and compared the calculated tumor content with that of the tumor specimen obtained by IHC for validation

**Fig. 3:**
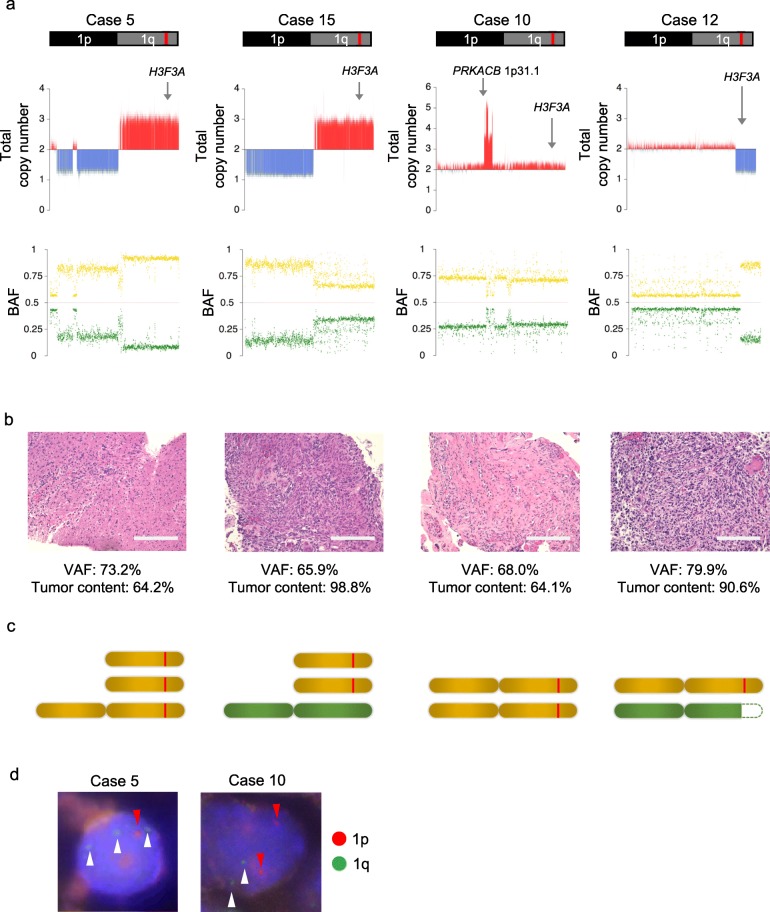
MASI of *H3F3A K27M* mutation determined with whole-genome sequencing. **a** Total copy number analyses of chromosome 1 on which *H3F3A* gene located (upper) and B allele frequency (BAF) of common SNPs (lower) with whole-genome sequencing (WGS) of four more than 50% VAF cases (cases 5, 15, 10, and 12). The red line shows the *H3F3A* gene region. The x-axis shows the gene location and the y-axis shows the copy number. Gray arrows indicate the *H3F3A* gene region and *PRKACB* gene region. Red and blue bars indicate gain and loss, respectively (upper). Higher or lower values between the moving average of the mismatch ratio of SNPs and (1-moving average of mismatch ratio of SNPs) are shown as a yellow or green dot, respectively (lower). **b** Hematoxylin-Eosin staining of tumor specimen (case 5, 15, 10, and 12; bars: 200um). In each case, the tumor content was calculated as H3 K27M positive cell numbers/DAPI positive cell numbers. **c** Expected chromosomal structure models of chromosome 1 in cases 5, 15, 10, and 12. The red line indicates the *H3F3A K27M* mutation. **d** FISH analysis of chromosome 1p and 1q in MASI cases (case 5; left and case 10; right). Red arrow heads indicated 1p36 signals (red) and white arrow heads indicated 1q25 signals (green) in DAPI-stained nuclei

In the four MASI cases included in the present study, we analyzed the copy number of all the chromosomes meticulously using WGS (Additional file 1: Figure S1). For chromosome 1, where the *H3F3A* gene is located, we also analyzed the BAF of the common SNPs. We evaluated the tumor content of the tumor specimen as the frequency of H3 K27M positive cells among DAPI positive cells. By combining these data with the VAF of the *H3F3A K27M* obtained by ddPCR, we determined the most appropriate chromosomal structure model for each case. Based on the total copy numbers of chromosome 1 obtained by WGS, we determined two candidate chromosome 1 copy number models exhibiting (i): copy number of integral value which is the closest to the mean value of copy number obtained by WGS and (ii): (i) + 1 in cases where copy number obtained by WGS was more than two, or (i) -1 in cases where copy number obtained by WGS was less than two. This is because normal cells (copy number: two) which were included in tumor tissue samples might decrease the calculated copy number value of tumor cells whose copy numbers were more than two, or increase that of tumor cells whose copy numbers were less than two. Next, in each candidate copy number model, we calculated the expected BAFs of the common SNPs on chromosome 1 using the tumor content of the tumor specimen. In these models, we selected two copy number models exhibiting the most consistent BAFs with those obtained by WGS. Next, we calculated the expected VAF of the *H3F3A K27M* in each selected candidate model using the tumor content of the tumor specimen. Then, we identified the most appropriate chromosomal structure model exhibiting more consistent expected VAF with that obtained by ddPCR in the two candidate models. Finally, we calculated the expected tumor content using the VAF of the *H3F3A K27M* obtained by ddPCR in this model and compared the expected tumor content with that of the tumor specimen for validation (Fig. 2). For cases 5 and 15, WGS revealed candidate models that exhibited an one copy loss of 1p and one or two copy gains of 1q in tumor cells (Fig. 3a). For case 5, we calculated the expected BAF of SNPs using the tumor content of the tumor specimen (64.2%; Fig. 3b). Then, we selected two models that exhibited the most consistent expected BAF of SNPs (86.4 and 89.1%) with that obtained by WGS (91.0%; Fig. 3a). Next, we calculated the expected VAF of the *H3F3A K27M* in these models (72.9 and 78.2%) using the tumor content of the tumor specimen (64.2%; Fig. 3b). We selected the model exhibiting the more consistent expected VAF of *H3F3A K27M* (72.9%) with that obtained by ddPCR (73.2%). We found that the chromosomal structure model that exhibited three *H3F3A K27M* mutant alleles without the wild-type allele was the most appropriate (Fig. 3c and Additional file 2: Figure S2). For case 15, we selected the two models that exhibited the most consistent BAF of SNPs (66.5 and 74.8%) calculated using the tumor content of the tumor specimen (98.8%; Fig. 3b) with that obtained by WGS (66.0%; Fig. 3a). We selected the chromosomal structure model that exhibited the more consistent VAF of the *H3F3A* K27M mutant (66.1%) calculated using the tumor content of the tumor specimen (98.8%; Fig. 3b) with that obtained by ddPCR (65.9%). We found that the chromosomal structure model exhibiting two *H3F3A K27M* mutant alleles with one wild-type allele (a total of three copies) was the most appropriate (Fig. 3c and Additional file 3: Figure S3). For case 10, the WGS indicated two candidate models that exhibited two or three copies of the whole chromosome 1 with focal amplification including the *PRKACB* gene, whose amplification in pediatric high-grade glioma has been reported previously [20] (Fig. 3a). We calculated the expected BAF of SNPs using the tumor content of the tumor specimen (64.1%; Fig. 3b) in each model. We selected two models that exhibited the most consistent expected BAF of SNPs (62.1 and 82.1%) with that obtained by the WGS (71.0%; Fig. 3a). In these models, the chromosomal structure model of two mutant alleles without a wild-type allele showed the more consistent expected VAF of *H3F3A K27M* (64.1%) with those obtained by ddPCR (68.0%) than model of two mutant alleles with a wild-type allele did (Fig. 3c and Additional file 4: Figure S4). For case 12, WGS revealed two candidate models that exhibited one or two copy regions on 1q around which the *H3F3A* gene was located in tumor cells (Fig. 3a). Based on the tumor content of the tumor specimen (90.6%; Fig. 3b), we calculated the expected BAF of the common SNPs in each model and found that these two models exhibited consistent BAF (91.4 and 95.3%) with that obtained by WGS (85.0%; Fig. 3a). In these models, we calculated the expected VAF of *H3F3A K27M* (82.8 and 90.6%) using the tumor content of the tumor specimen (90.6%; Fig. 3b). Then, we selected the chromosomal structure model that exhibited the more consistent VAF of *H3F3A K27M* (82.8%) with those obtained by ddPCR (79.9%). The most appropriate chromosomal structure model of this case was the partial loss of 1q containing the wild-type *H3F3A* allele (Fig. 3c and Additional file 5: Figure S5). In all these cases, we confirmed that the calculated tumor contents based on the most appropriate chromosomal structure model were consistent with those of the tumor specimen (Additional file 2: Figure S2, Additional file 3: Figure S3, Additional file 4: Figure S4 and Additional file 5: Figure S5). These data revealed that all four cases exhibited MASI of *H3F3A K27M* mutant with (cases 5,10, and 12) or without (case 15) loss of the wild-type allele (Fig. 3c). We also found consistent copy number variations on chromosome 1p and 1q in all MASI cases using the FISH assay (Fig. 3d), although FISH assay also revealed slight heterogeneous copy number variations on chromosome 1p and 1q in these cases.

### Aggressive phenotype in cases exhibiting MASI of H3F3A K27M

**Fig. 4:**
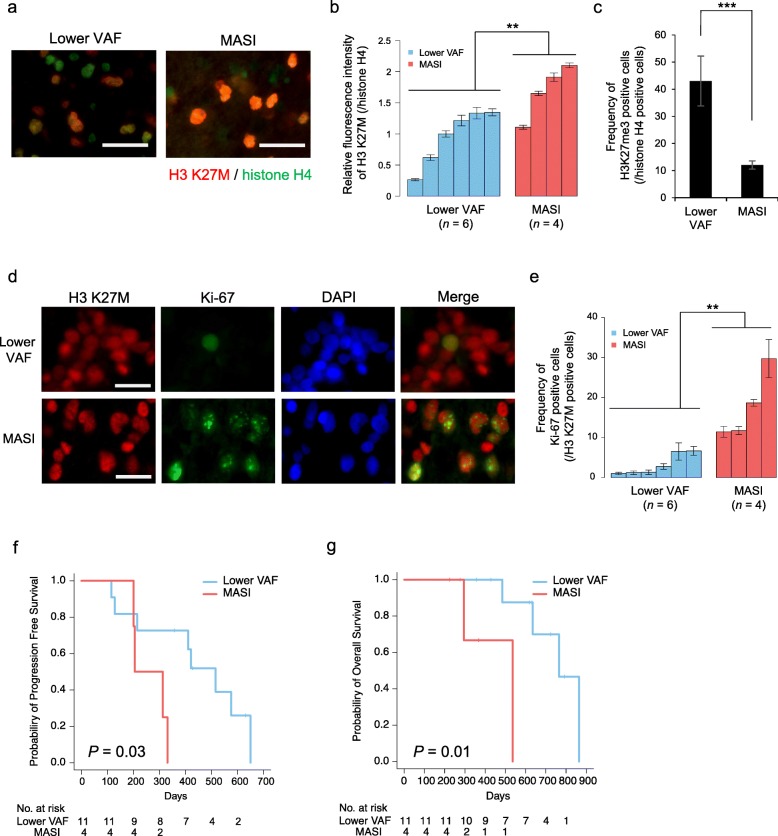
Aggressive phenotype in cases exhibiting MASI of *H3F3A K27M*. **a** Immunofluorescence staining of H3 K27M (red) and histone H4 (green) as an internal control of MASI case (case 5; bars, 30 μm) and lower VAF case (case 6). **b** Bar graph of relative fluorescence intensity of H3 K27M to those of histone H4 in lower VAF and MASI cases (*n* = 6 and *n* = 4, respectively). Error bars indicate the standard deviation (SD) (**P < 0.05). (c) Bar graph indicates the frequency of H3K27me3 positive cells among histone H4 positive cells in lower VAF and MASI cases (*n* = 6 and *n* = 4, respectively). Error bars indicate the SD (***P < 0.01). **d** Immunofluorescence staining of Ki-67 (green), H3 K27M (red), and DAPI (blue) using the tumor specimen of the MASI case (case 5; bars, 20 μm) and lower VAF case (case 4). **e** Bar graphs indicating the frequency of Ki-67 positive cells among H3 K27M positive cells in lower VAF cases (*n* = 6, blue) and MASI cases (*n* = 4, red). Error bars indicate the SD (**P < 0.05). **f** Kaplan-Meier survival curves illustrating the progression-free survival (PFS) of MASI cases (*n* = 4) and lower VAF cases (*n* = 11; *P* = 0.03). **g** Kaplan-Meier survival curves illustrating the overall survival (OS) of MASI cases (*n* = 4) and lower VAF cases (*n* = 11; *P* = 0.01)

During tumor formation, H3 K27M plays a pivotal role via the global reduction of H3K27me3 modification. Therefore, we investigated the expression level of H3 K27M and H3K27me3 in MASI of *H3F3A K27M* cases with IHC. IHC revealed that the MASI cases (*n* = 4) exhibited a significantly higher expression level of H3 K27M and lower level of H3K27me3 than those in cases with lower VAF (*n* = 6) (*P* < 0.05 and *P* < 0.01, respectively) (Fig. 4a–c, Table 1) together with comparable tumor cellularity between lower VAF cases and MASI cases (mean: 77.0 and 79.4%, respectively). We validated these findings using Western blot analysis in the two available cases (Additional file 6: Figure S6). Additionally, the four MASI cases showed a significantly higher Ki-67 index than that in the lower VAF cases (*P* < 0.05) (Fig. 4d and e). These data revealed that the MASI of *H3F3A K27M* mutation was associated with promoted cell proliferation, which might be caused via the upregulation of H3 K27M expression. Next, we investigated the characteristic clinical features of the MASI cases. Compared with the lower VAF cases (*n* = 11), MASI cases (*n* = 4) did not exhibit characteristic clinical features such as age and tumor location; however, MASI cases exhibited a significantly poorer PFS (*P* = 0.03; Fig. 4f) and OS (*P* = 0.01; Fig. 4g) compared with the lower VAF cases. *TP53* and *ATRX* gene mutations were previously reported to be the most frequently found gene mutations in diffuse midline glioma, H3 K27M-mutant cases [21]. In our case, we also found *TP53* gene mutations (*n* = 3; case 5, 12 and 14) and *ATRX* gene mutations (*n* = 3; case 2, 7 and 12) however, these gene mutations were not significantly correlated with poorer prognosis and followed the same correlation even in the presence of MASI. These data revealed that the MASI of *H3F3A K27M* was associated with the aggressive phenotype of diffuse midline glioma, H3 K27M-mutant.

## Discussion

In the present study, we found that several diffuse midline glioma, H3 K27M-mutant exhibited higher VAF than 50% obtained by ddPCR assay. To the best of our knowledge, our study is the first study to identify the MASI of *H3F3A K27M* cases in these types of glioma by WGS analyses. MASI cases exhibited a significantly higher Ki-67 index in the tumor specimens and poorer survival than those of lower VAF cases; therefore, MASI was associated with an aggressive phenotype of this type of glioma. H3 K27M is known to inhibit the methyltransferase activity of PRC2 that catalyzes the tri-methylation of H3K27, resulting in a reduction in the overall H3K27me3 level [22, 23]. Consistently, our study revealed that the MASI cases exhibited significantly lower H3K27me3 levels together with higher expression levels of H3 K27M compared with those in the lower VAF cases. In the human colorectal cancer cell lines, the MASI of the *KRAS* gene mutation has been reported to be sensitive to MEK inhibitors [12]. These data indicated that upregulated H3 K27M might be a potent treatment target for diffuse midline glioma, H3 K27M-mutant exhibiting MASI of *H3F3A K27M*.

There were previous studies where *KRAS* MASI elevated *KRAS* mRNA levels, increasing RAS activity [14, 24] and wild-type allele of *KRAS* has also been shown to play a pivotal role as a tumor suppressor [25]. Even in cases exhibiting a conventional heterozygous mutation of *H3F3A* gene genetically, downregulation of wild-type *H3F3A* via epigenetic dysregulation might contribute to the aggressive phenotype of this type of glioma, although further investigation is required.

MASI was considered to play a pivotal role in obtaining a selective growth advantage by increasing and tuning the dosage of mutations of oncogenic driver genes in cancer cells. The modest gain of the mutant allele or loss of the wild-type allele enhanced fitness and contributed to clonal outgrowth in most oncogenes [11]. These findings suggested that a subset of tumor cells exhibiting heterozygous mutation of *H3F3A K27M* might acquire MASI of *H3F3A K27M* during tumor formation of an aggressive subtype of diffuse midline glioma, H3 K27M-mutant. In the present study, we demonstrated that the MASI of *H3F3A K27M* mutation was associated with the aggressive phenotype via upregulation of H3 K27M together with downregulation of H3K27me3 in this type of glioma, resulting in a poor prognosis. Further analysis in a larger cohort may reinforce our conclusion because our total sample numbers were small (*n* = 15). Further investigation of the molecular mechanisms by which MASI of *H3F3A K27M* is associated with the aggressive phenotype of these cases might contribute to elucidating the tumor formation mechanism of an aggressive subset of diffuse midline glioma, H3 K27M-mutant.

## Conclusion

DdPCR and WGS analyses revealed that the copy number gain of the mutant allele and/or loss of the wild-type allele of *H3F3A* gene constituted MASI of *H3F3A K27M* in diffuse midline glioma, H3 K27M-mutant. MASI of *H3F3A K27M* mutation was closely associated with an aggressive subtype of diffuse midline glioma, H3 K27M-mutant.

## Supplementary information

**Additional file 1: Figure S1.** Copy numbers of whole chromosomes in four MASI cases. Total copy number of whole chromosomes obtained by WGS in four MASI cases (case 5, 10, 12 and 15). Case 5, 10 and 15 were male (X: one and Y: one). Case 12 was female (X: two).

**Additional file 2: Figure S2.** Flowchart indicating identification of the most appropriated chromosomal structure model in case 5. Total copy number of 1q obtained by WGS (3 ≦), tumor content in tumor specimen (64.2%), BAF of SNPs obtained by WGS (91.0%), and VAF of *H3F3A K27M* obtained by ddPCR (73.2%) were used to reveal the most appropriate model of the 1q arm of tumor cells. The calculated tumor content with VAF of *H3F3A K27M* in the most appropriate model (64.6%) was consistent with that of the tumor specimen (64.2%).

**Additional file 3: Figure S3.** Flowchart indicating identification of the most appropriated chromosomal structure model in case 15. Total copy number of 1q obtained by WGS (3 ≦), tumor content in tumor specimen (98.8%), BAF of SNPs obtained by WGS (66.0%), and VAF of *H3F3A K27M* obtained by ddPCR (65.9%) were used to reveal the most appropriate model of 1q arm of tumor cells. The calculated tumor content with VAF of *H3F3A K27M* in the most appropriate model (98.3%) was consistent with that of the tumor specimen (98.8%).

**Additional file 4: Figure S4.** Flowchart indicating identification of the most appropriate chromosomal structure model in case 10. Total copy number of 1q obtained by WGS (2 ≦), tumor content in tumor specimen (64.1%), BAF of SNPs obtained by WGS (71.0%), and VAF of *H3F3A K27M* obtained by ddPCR (68.0%) were used to reveal the most appropriate model of 1q arm of tumor cells. The calculated tumor content with VAF of *H3F3A K27M* in the most appropriate model (68.0%) was consistent with that of the tumor specimen (64.1%).

**Additional file 5: Figure S5.** Flowchart indicating identification of the most appropriate chromosomal structure model in case 12. Total copy number of 1q obtained by WGS (2 ≧), tumor content in tumor specimen (90.6%), BAF of SNPs obtained by WGS (85.0%), and VAF of *H3F3A K27M* obtained by ddPCR (79.9%) were used to reveal the most appropriate model of 1q arm of tumor cells. The calculated tumor content with VAF of *H3F3A K27M* in the most appropriate model (88.8%) was consistent with that in of the tumor specimen (90.6%).

**Additional file 6: Figure S6.** Protein expression levels of H3 K27M and H3K27me3. Protein expression level of H3 K27M (upper) and H3K27me3 (lower) in lower VAF case (case 6) and MASI case (case 10). *H3F3A* wild-type gliomas (*IDH1* mutated) sample was used for a negative control (NC) for H3 K27M and a positive control (PC) for H3K27me3. Histone H3 protein expression level was used as an internal control.

## Data Availability

The datasets generated during the current study are available in the National Bioscience Database Center (NBDC) repository (https://biosciencedbc.jp/en/).

## Abbreviations

BAF: B Allele Frequency
ddPCR: Droplet Digital Polymerase Chain Reaction
DIPG: Diffuse Intrinsic Pontine Glioma
FFPE: Formalin-Fixed Paraffin-Embedded
FISH: Fluorescence in situ hybridization
IHC: Immunohistochemistry
MASI: Mutant Allele Specific Imbalance
OS: Overall Survival
PFS: Progression-Free Survival
SD: Standard Deviation
SNPs: Single Nucleotide Polymorphisms
VAF: Variant Allele Frequency
WGS: Whole-Genome Sequencing
