# Supplementary figures and images for: H3F3A mutant allele specific imbalance in an aggressive subtype of diffuse midline glioma, H3 K27M-mutant
Source: Acta Neuropathol Commun. 2020 Feb 5;8:8. doi: 10.1186/s40478-020-0882-4 (PMC7001313; doi:10.1186/s40478-020-0882-4)

## Slide 1
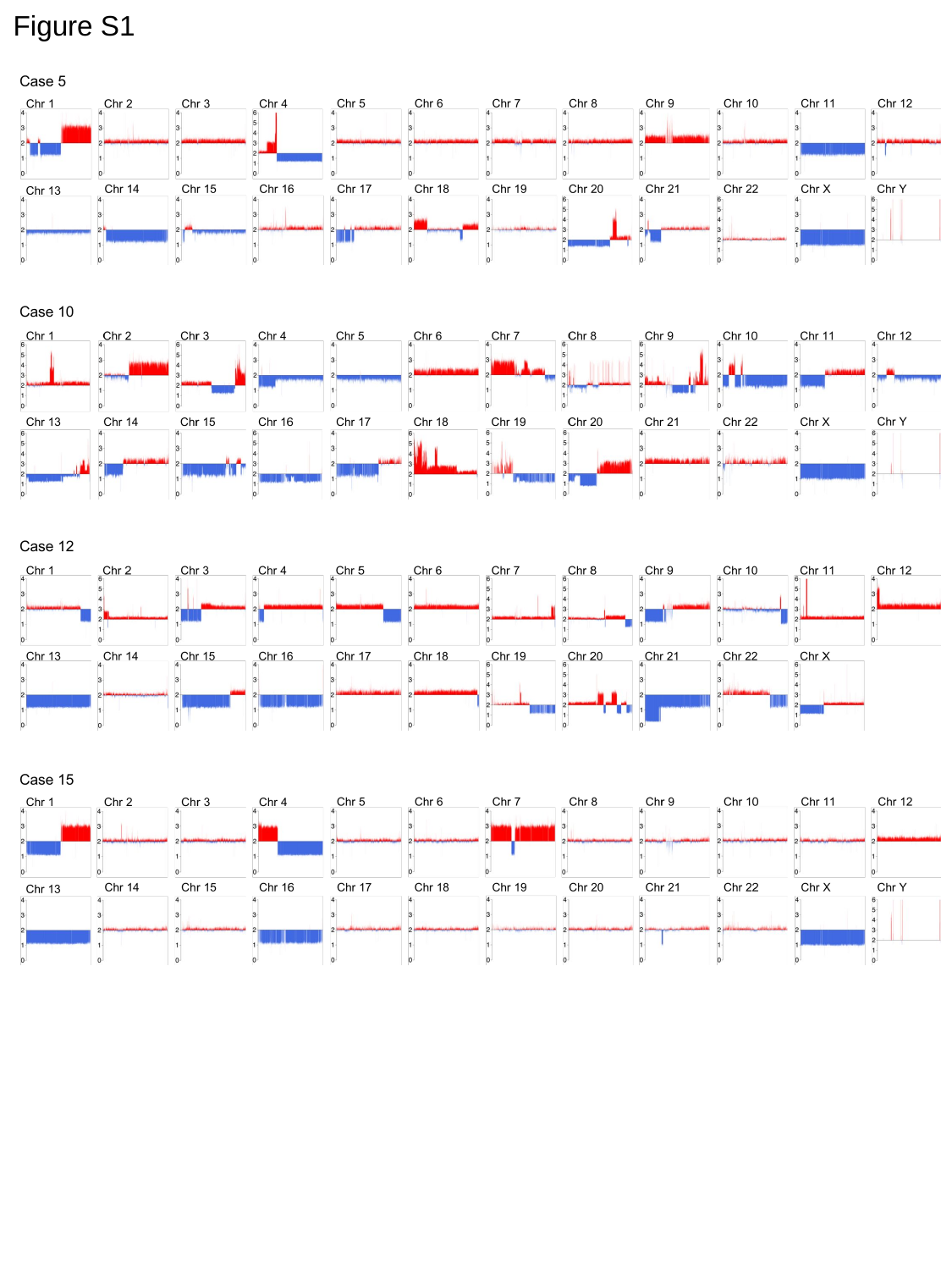

Figure S1

Supplement: Supplementary file 1 — Additional file 1: Figure S1. Copy numbers of whole chromosomes in four MASI cases. Total copy number of whole chromosomes obtained by WGS in four MASI cases (case 5, 10, 12 and 15). Case 5, 10 and 15 were male (X: one and Y: one). Case 12 was female (X: two). [file 40478_2020_882_MOESM1_ESM.pptx]

## Slide 1
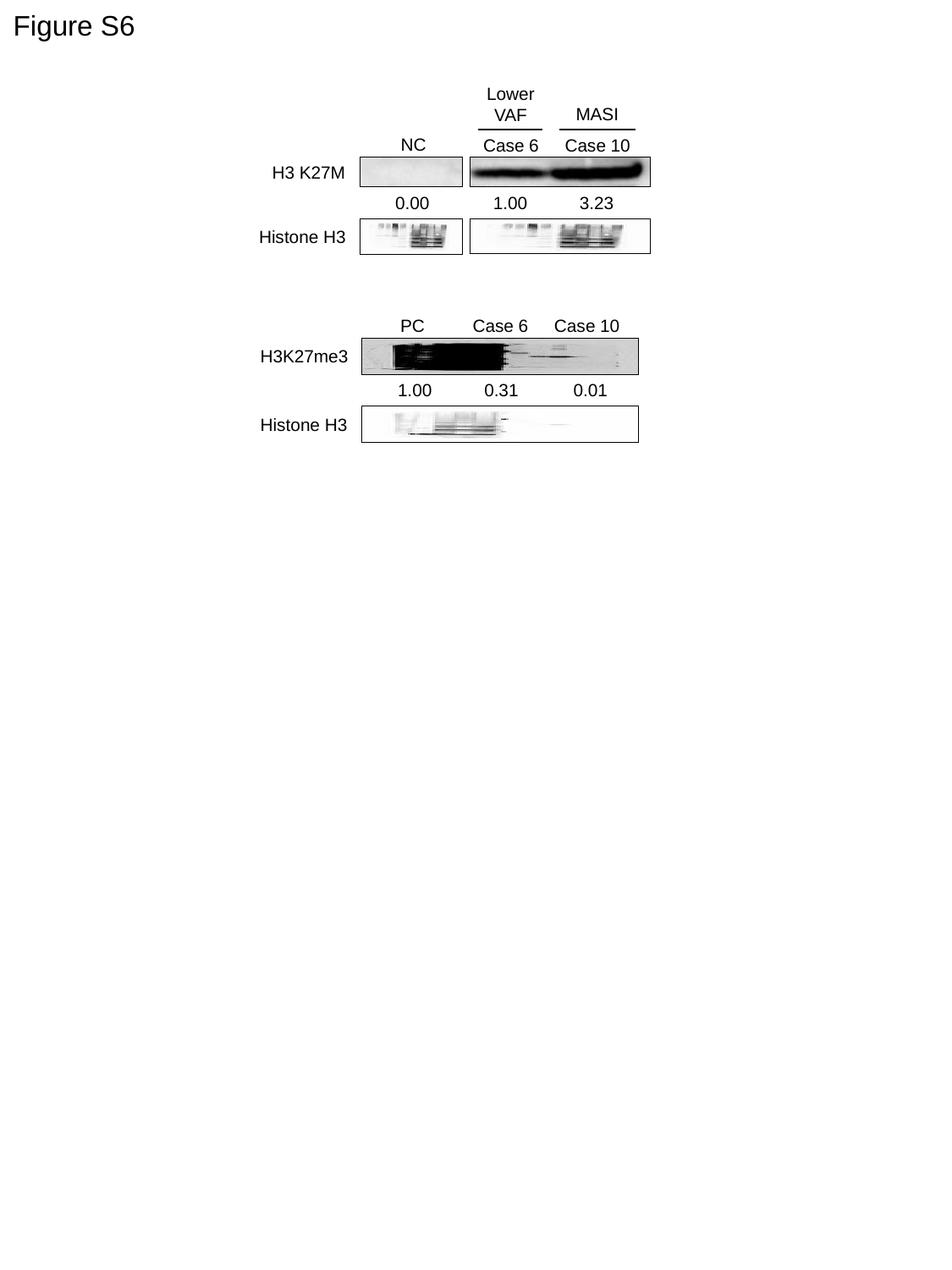

Figure S6
Lower
VAF
MASI
NC
Case 6
Case 10
H3 K27M
1.00
3.23
0.00
Histone H3
Case 6
Case 10
PC
H3K27me3
1.00
0.31
0.01
Histone H3

Supplement: Supplementary file 6 — Additional file 6: Figure S6. Protein expression levels of H3 K27M and H3K27me3. Protein expression level of H3 K27M (upper) and H3K27me3 (lower) in lower VAF case (case 6) and MASI case (case 10). H3F3A wild-type gliomas (IDH1 mutated) sample was used for a negative control (NC) for H3 K27M and a positive control (PC) for H3K27me3. Histone H3 protein expression level was used as an internal control. [file 40478_2020_882_MOESM6_ESM.pptx]
