# Supplementary material for: H3F3A mutant allele specific imbalance in an aggressive subtype of diffuse midline glioma, H3 K27M-mutant
Source: Acta Neuropathol Commun. 2020 Feb 5;8:8. doi: 10.1186/s40478-020-0882-4 (PMC7001313; doi:10.1186/s40478-020-0882-4)
Supplement: Supplementary file 2 — Additional file 2: Figure S2. Flowchart indicating identification of the most appropriated chromosomal structure model in case 5. Total copy number of 1q obtained by WGS (3 ≦), tumor content in tumor specimen (64.2%), BAF of SNPs obtained by WGS (91.0%), and VAF of H3F3A K27M obtained by ddPCR (73.2%) were used to reveal the most appropriate model of the 1q arm of tumor cells. The calculated tumor content with VAF of H3F3A K27M in the most appropriate model (64.6%) was consistent with that of the tumor specimen (64.2%). [file 40478_2020_882_MOESM2_ESM.pptx]

## Slide 1
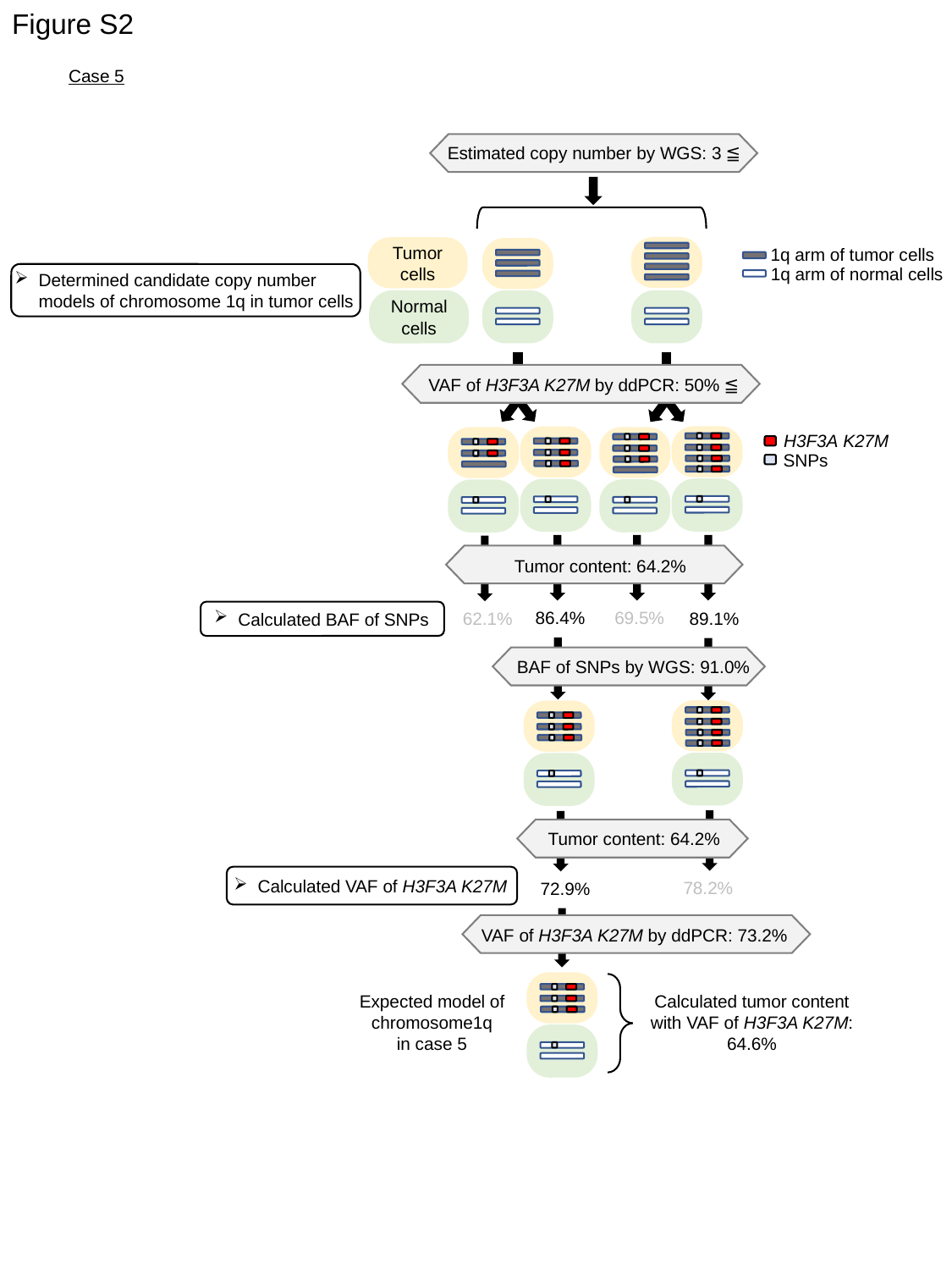

Figure S2
Case 5
Estimated copy number by WGS: 3 ≦
1q arm of tumor cells
Tumor cells
1q arm of normal cells
Determined candidate copy number models of chromosome 1q in tumor cells
Normal cells
VAF of H3F3A K27M by ddPCR: 50% ≦
H3F3A K27M
SNPs
Tumor content: 64.2%
69.5%
86.4%
62.1%
89.1%
Calculated BAF of SNPs
BAF of SNPs by WGS: 91.0%
Tumor content: 64.2%
Calculated VAF of H3F3A K27M
78.2%
72.9%
VAF of H3F3A K27M by ddPCR: 73.2%
Expected model of chromosome1q
in case 5
Calculated tumor content with VAF of H3F3A K27M:
64.6%
