# Supplementary material for: H3F3A mutant allele specific imbalance in an aggressive subtype of diffuse midline glioma, H3 K27M-mutant
Source: Acta Neuropathol Commun. 2020 Feb 5;8:8. doi: 10.1186/s40478-020-0882-4 (PMC7001313; doi:10.1186/s40478-020-0882-4)
Supplement: Supplementary file 3 — Additional file 3: Figure S3. Flowchart indicating identification of the most appropriated chromosomal structure model in case 15. Total copy number of 1q obtained by WGS (3 ≦), tumor content in tumor specimen (98.8%), BAF of SNPs obtained by WGS (66.0%), and VAF of H3F3A K27M obtained by ddPCR (65.9%) were used to reveal the most appropriate model of 1q arm of tumor cells. The calculated tumor content with VAF of H3F3A K27M in the most appropriate model (98.3%) was consistent with that of the tumor specimen (98.8%). [file 40478_2020_882_MOESM3_ESM.pptx]

## Slide 1
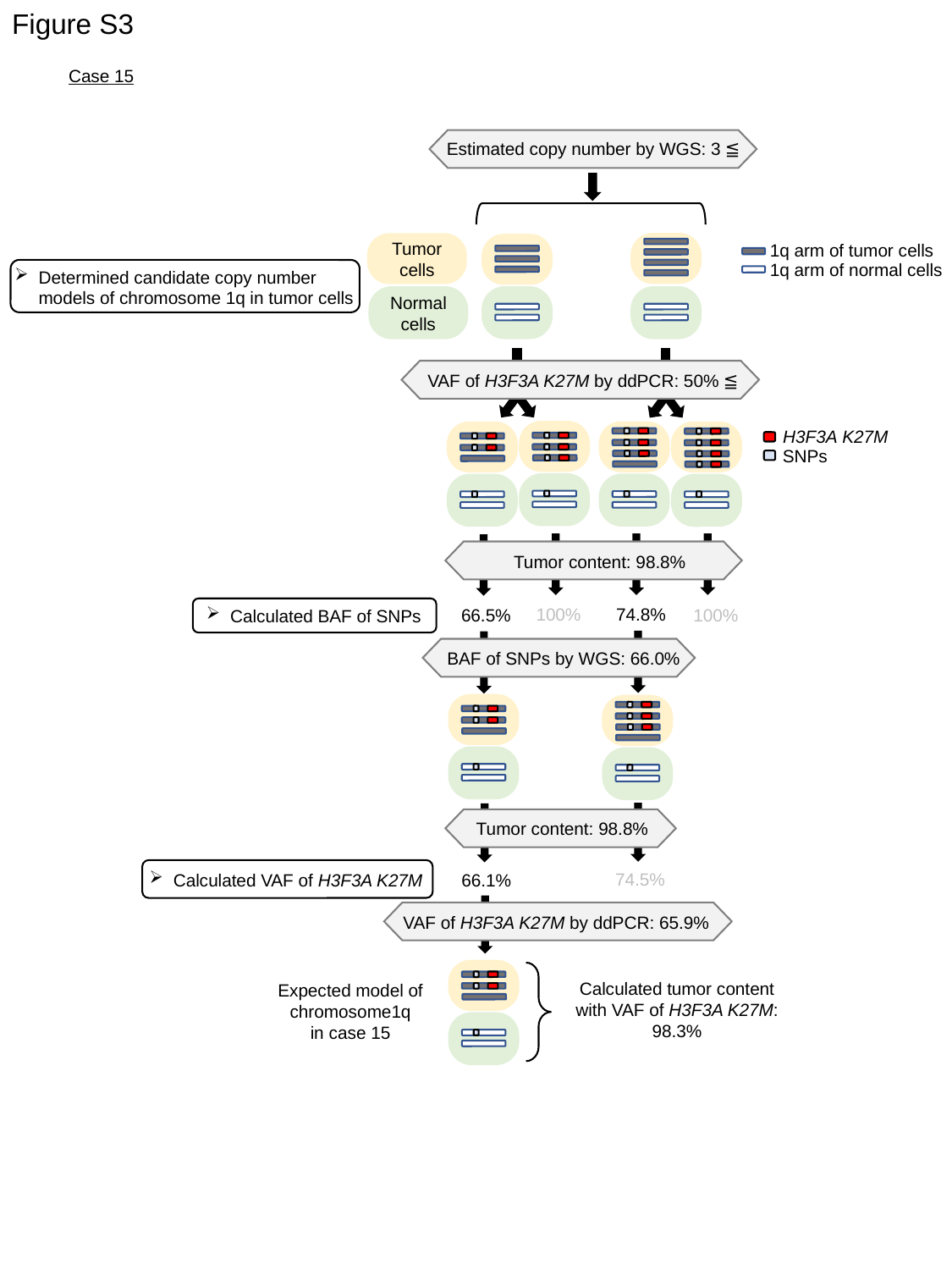

Figure S3
Case 15
Estimated copy number by WGS: 3 ≦
1q arm of tumor cells
Tumor cells
1q arm of normal cells
Determined candidate copy number models of chromosome 1q in tumor cells
Normal cells
VAF of H3F3A K27M by ddPCR: 50% ≦
H3F3A K27M
SNPs
Tumor content: 98.8%
74.8%
100%
66.5%
100%
Calculated BAF of SNPs
BAF of SNPs by WGS: 66.0%
Tumor content: 98.8%
Calculated VAF of H3F3A K27M
74.5%
66.1%
VAF of H3F3A K27M by ddPCR: 65.9%
Calculated tumor content with VAF of H3F3A K27M:
98.3%
Expected model of chromosome1q
in case 15
