# Supplementary material for: H3F3A mutant allele specific imbalance in an aggressive subtype of diffuse midline glioma, H3 K27M-mutant
Source: Acta Neuropathol Commun. 2020 Feb 5;8:8. doi: 10.1186/s40478-020-0882-4 (PMC7001313; doi:10.1186/s40478-020-0882-4)
Supplement: Supplementary file 4 — Additional file 4: Figure S4. Flowchart indicating identification of the most appropriate chromosomal structure model in case 10. Total copy number of 1q obtained by WGS (2 ≦), tumor content in tumor specimen (64.1%), BAF of SNPs obtained by WGS (71.0%), and VAF of H3F3A K27M obtained by ddPCR (68.0%) were used to reveal the most appropriate model of 1q arm of tumor cells. The calculated tumor content with VAF of H3F3A K27M in the most appropriate model (68.0%) was consistent with that of the tumor specimen (64.1%). [file 40478_2020_882_MOESM4_ESM.pptx]

## Slide 1
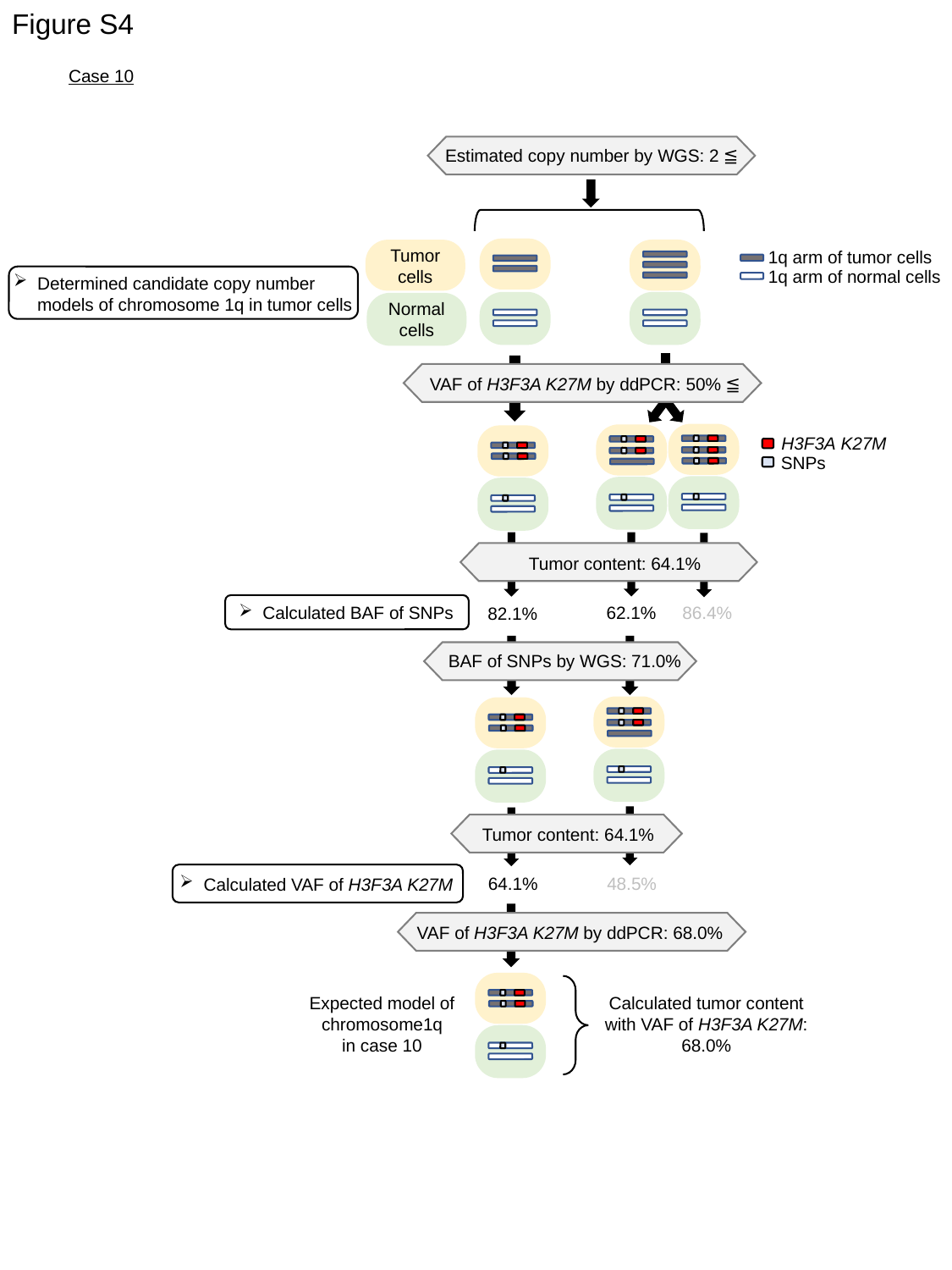

Figure S4
Case 10
Estimated copy number by WGS: 2 ≦
1q arm of tumor cells
Tumor cells
1q arm of normal cells
Determined candidate copy number models of chromosome 1q in tumor cells
Normal cells
VAF of H3F3A K27M by ddPCR: 50% ≦
H3F3A K27M
SNPs
Tumor content: 64.1%
Calculated BAF of SNPs
86.4%
62.1%
82.1%
BAF of SNPs by WGS: 71.0%
Tumor content: 64.1%
Calculated VAF of H3F3A K27M
48.5%
64.1%
VAF of H3F3A K27M by ddPCR: 68.0%
Calculated tumor content with VAF of H3F3A K27M:
68.0%
Expected model of chromosome1q
in case 10
