# Supplementary material for: H3F3A mutant allele specific imbalance in an aggressive subtype of diffuse midline glioma, H3 K27M-mutant
Source: Acta Neuropathol Commun. 2020 Feb 5;8:8. doi: 10.1186/s40478-020-0882-4 (PMC7001313; doi:10.1186/s40478-020-0882-4)
Supplement: Supplementary file 5 — Additional file 5: Figure S5. Flowchart indicating identification of the most appropriate chromosomal structure model in case 12. Total copy number of 1q obtained by WGS (2 ≧), tumor content in tumor specimen (90.6%), BAF of SNPs obtained by WGS (85.0%), and VAF of H3F3A K27M obtained by ddPCR (79.9%) were used to reveal the most appropriate model of 1q arm of tumor cells. The calculated tumor content with VAF of H3F3A K27M in the most appropriate model (88.8%) was consistent with that in of the tumor specimen (90.6%). [file 40478_2020_882_MOESM5_ESM.pptx]

## Slide 1
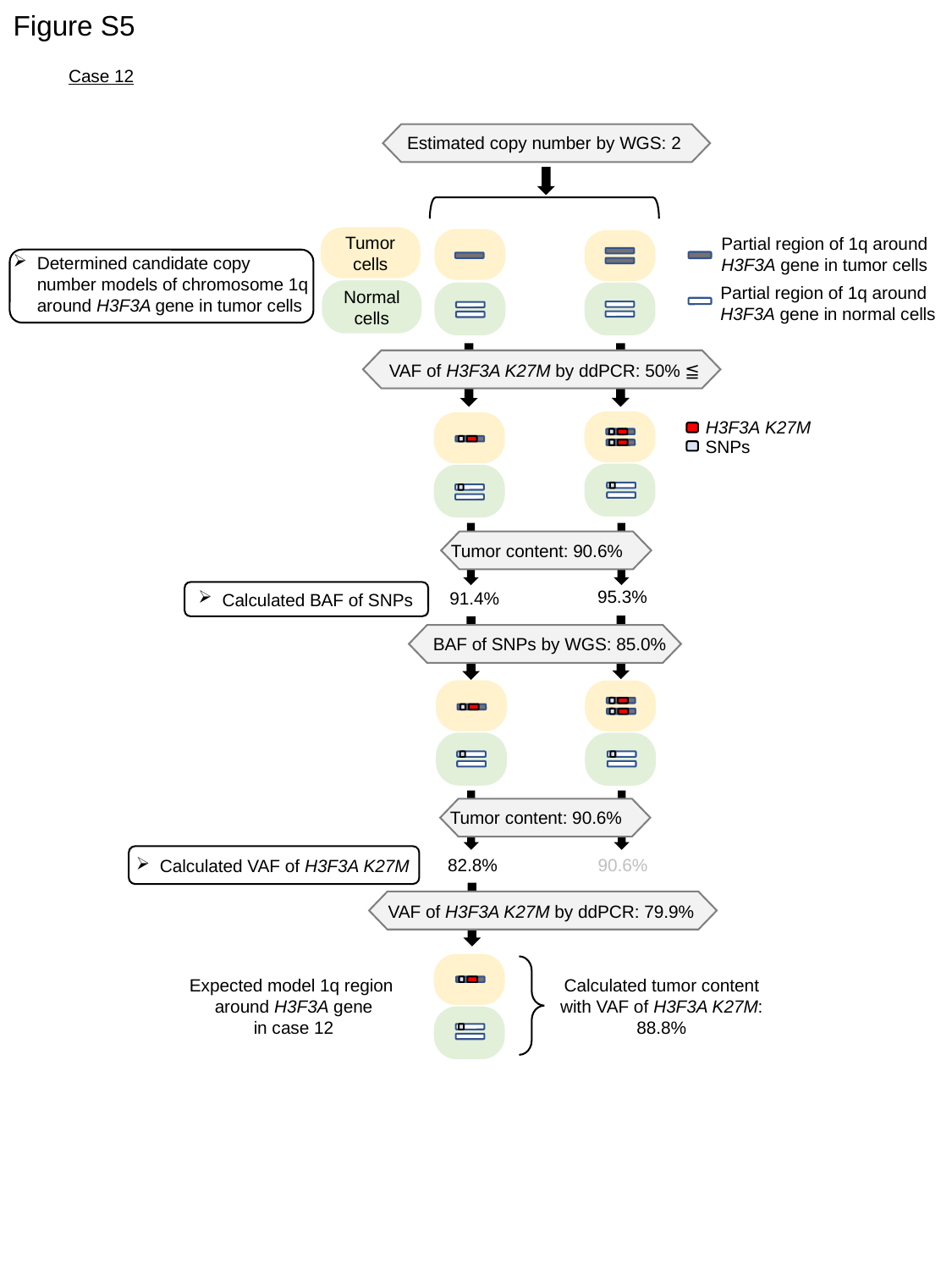

Figure S5
Case 12
Partial region of 1q around H3F3A gene in tumor cells
Tumor cells
Determined candidate copy number models of chromosome 1q around H3F3A gene in tumor cells
Partial region of 1q around H3F3A gene in normal cells
Normal cells
VAF of H3F3A K27M by ddPCR: 50% ≦
H3F3A K27M
SNPs
Tumor content: 90.6%
95.3%
91.4%
Calculated BAF of SNPs
BAF of SNPs by WGS: 85.0%
Tumor content: 90.6%
Calculated VAF of H3F3A K27M
82.8%
90.6%
VAF of H3F3A K27M by ddPCR: 79.9%
Calculated tumor content with VAF of H3F3A K27M:
88.8%
Expected model 1q region
around H3F3A gene
in case 12
